# The germline/somatic DNA damage repair gene mutations modulate the therapeutic response in Chinese patients with advanced pancreatic ductal adenocarcinoma

**DOI:** 10.1186/s12967-021-02972-6

**Published:** 2021-07-12

**Authors:** Lin Shui, Xiaofen Li, Yang Peng, Jiangfang Tian, Shuangshuang Li, Du He, Ang Li, Bole Tian, Mao Li, Heli Gao, Ning An, Cheng Yi, Dan Cao

**Affiliations:** 1 Department of Abdominal Oncology, Cancer Center, State Key Laboratory of Biotherapy, West China Hospital, Sichuan University, Chengdu, China; 2 Department of Oncology Radiation and Chemotherapy, West China Second University Hospital, Sichuan University, Chengdu, China; 3 Department of Breast Surgery, The First Affiliated Hospital of Chongqing Medical University, Chongqing, China; 4 Department of Oncology, The Second Affiliated Hospital of Chengdu Medical College, China National Nuclear Corporation 416 Hospital, Chengdu, China; 5 Department of pathology, West China Hospital, Sichuan University, Chengdu, China; 6 Pancreatic Surgery, West China Hospital, Sichuan University, Chengdu, China; 7 Department of Oncology, the Cancer Hospital of Fudan University, Shanghai, China; 8 Department of oncology, the People’s Hospital of Sichuan Province, Chengdu, China

**Keywords:** Pancreatic ductal adenocarcinoma, DNA damage repair gene, Next generation sequencing, Chinese population

## Abstract

**Background:**

Pancreatic ductal adenocarcinoma (PDAC) is a fatal disease with molecular heterogeneity, inducing differences in biological behavior, and therapeutic strategy. NGS profiles of pathogenic alterations in the Chinese PDAC population are limited. We conducted a retrospective study to investigate the predictive role of DNA damage repair (DDR) mutations in precision medicine.

**Methods:**

The NGS profiles were performed on resected tissues from 195 Chinese PDAC patients. Baseline clinical or genetic characteristics and survival status were collected. The Kaplan–Meier survival analyses were performed by the R version 3.6.1.

**Results:**

The main driver genes were KRAS, TP53, CDKN2A, and SMAD4. Advanced patients with KRAS mutation showed a worse OS than KRAS wild-type (p = 0.048). DDR pathogenic deficiency was identified in 30 (15.38%) of overall patients, mainly involving BRCA2 (n = 9, 4.62%), ATM (n = 8, 4.10%) and RAD50 genes (n = 3, 1.54%). No significance of OS between patients with or without DDR mutations (p = 0.88). But DDR mutation was an independent prognostic factor for survival analysis of advanced PDAC patients (p = 0.032). For DDR mutant patients, treatment with platinum-based chemotherapy (p = 0.0096) or olaparib (p = 0.018) respectively improved the overall survival. No statistical difference between tumor mutation burden (TMB) and DDR mutations was identified. Treatment of PD-1 blockades did not bring significantly improved OS to DDR-mutated patients than the naive DDR group (p = 0.14).

**Conclusions:**

In this retrospective study, we showed the role of germline and somatic DDR mutation in predicting the efficacy of olaparib and platinum-based chemotherapy in Chinese patients. However, the value of DDR mutation in the prediction of hypermutation status and the sensitivity to the PD-1 blockade needed further investigation.

**Supplementary Information:**

The online version contains supplementary material available at 10.1186/s12967-021-02972-6.

## Background

Between 2009 and 2016, the five-year survival of pancreatic ductal adenocarcinoma (PDAC) fluctuated less than 9% [1]. The reasons for the high mortality of PDAC primarily include the insidious onset, fast-growing invasion, and ineffective treatment [2]. The standard of care was limited to gemcitabine in metastatic settings. Novel cytotoxic agents and cell signaling inhibitors hardly improve clinical outcomes [3]. Given the increasing incidence of PDAC, there is a major unconquered challenge to develop more effective therapeutic strategies.

Deficiency might render a PDAC vulnerable to a potential new therapeutic intervention that increases the DNA damage load beyond a tolerable threshold [4]. Therapeutic strategies targeting DDR pathways are widely used in anti-tumor treatments [5]. For example, poly (ADP-ribose) polymerase (PARP) inhibitors used in BRCA mutated patients may lead to disruption of two redundant DDR pathways and accumulation of DNA damages [6], thus presenting the phenomenon of synthetic lethality and triggering the apoptosis or necroptosis of tumor cells [7]. Platinum is a chemotherapeutic agent to cross-link purines on DNA and cause DNA damages. Theoretically, these DNA breaks induced by PARP inhibitors or platinum cannot be effectively repaired when DDR genes are mutated. Another indirect DNA repair-related therapy is an immune checkpoint inhibitor (ICI) [8]. Compromised repair of DNA induces the accumulation of cytoplasmic DNA fragments, which may increase neoantigen load and immunogenicity. As a result, high-mutational status, such as a high level of tumor mutation burden and high expression of PD-L1, may result in high sensitivity to immunotherapy, especially ICIs [9].

Tumor genetic profiling may help determine optimal treatments. Most of the current large-scale studies involved PDAC patients from western countries. The prevalence of tumor driver genes and DDR deficiency in Chinese PDACs remains unknown, and the relationship between both germline and somatic DDR mutations and the survival or the sensitivity to relevant therapy is not clear. Previous studies have demonstrated the role of germline BRCA1/2 mutations in the prediction of the sensitivity to DNA damage targeting treatment [10, 11]. Currently, mounting evidence showed that DDR deficiency also occurred in sporadic PDACs, such as somatic BRCA1/2, ATM, RAD51 mutations [12, 13]. It was more comprehensive to take both germline and somatic mutations into consideration. Herein we conducted a study to demonstrate the mutation landscape of Chinese PDAC patients and explore the predictive role of germline and somatic DDR mutations in guiding the treatment strategies.

## Methods and material

### Study population and patient enrollment

Patients who were pathologically confirmed PDAC and were profiled by NGS of formalin-fixed paraffin-embedded tissue between January 2016 to November 2019 were eligible for our study. The majority of patients were admitted to West China Medical Center, except one patient from the People's Hospital of Sichuan Province and two patients from the Cancer Hospital of Fudan University. Exclusion criteria included final pathology other than PDAC, and patients who had less-than-one-month overall survival.

The study was approved by the Institutional Review Board of West China Medical Center with a waiver of informed consent. This was mainly because of the retrospective nature of the study and the fact that most patients had died at the time of study conception.

### Clinical characteristics collection

Baseline demographic, clinical, and pathologic information of enrolled patients were collected and recorded at the time of diagnosis. The definition of family history is these first degree relatives with a history of any solid malignancy. R1 was defined as a distance of tumor cells < 1 mm from the closest resection margin and R0 ≥ 1 mm. Platinum-based chemotherapy included the use of cisplatin or oxaliplatin. Patients were followed with a CT scan of the chest, abdomen, and pelvis every 3 months for the first half-year and 6 months once thereafter. Recurrence was defined as the imaging observation of distant metastases or progressing change within the surgical bed including the pancreas remnant or anastomosis sites. Limited stage referred to the resectable carcinoma that is still limited to the pancreas. Advanced disease was defined as a local infiltrated, unresectable, or metastatic lesion during the whole course of the disease. The date of diagnosis to the date of death or censored at the date of the last follow-up was collected for OS calculation.

### NGS profiling

Gene panels used in this study were designed to describe the critical gene mutations in solid tumors. A total of three gene panels were performed retrospectively in this study, including 150, 381 and 417 genes, respectively (3DMed Company). Methods and protocols of NGS profiling were described in detail as the previous article [14]. The NGS platform uses ILLUMINA Nextseq500 to perform whole-exome sequencing. Each of the bases in the genome is sequenced more than 800 times to deliver accurate data and insight into unexpected DNA variation.

The three gene panels provided a comprehensive genomic profile of 390, 150, and 417 genes in one single test, respectively. The detailed genes detected in these panels were provided in Additional file 1. The alterations of SNV, InDels, fusions/rearrangements, and amplification/loss were detected in these panels. From 10 to 50 main DDR-related genes were included in our NGS panel and the main genes of them including ATM, BRCA1/2, BLM, CHEK1, CHEK2, FANCA, FANCE, FANCD2, MLH1, MSH2, MSH6, PALB2, PMS2, RAD50, RAD51. The functional significances of variants in DDR genes were classified into the benign, likely benign, variant of uncertain significance (VOUS), likely pathogenic, and pathogenic variants according to the ACMG standard [15]. Pathogenic mutations were defined as those variants that would affect the function of a gene, including nonsense, frameshift, and splice-site mutations. The evidence for pathogenic mutations was mainly derived from public databases or published literature. Mutations with variants of unknown significance were excluded in this study. Germline variation referred to the heritable variation detected in blood samples whereas somatic mutation testing was done using tumor tissue.

Besides, the 417-gene panel also presented biomarkers related to immunotherapy, including tumor mutation load (TMB), PD-L1 expression, and the status of the microsatellite. TMB refers to the number of somatic mutations per million bases (Mb) in the targeted sequencing coding region, including point mutations and insertion deletions. TMB was classified as high, medium, and low according to the internal database of tumor species. The high level of TMB was defined as the range of top 25%, medium level as 26%–75%, and low as 76%–100%. PD-L1 expression on tumor cells was assessed by VENTANA PD-L1 (SP263) assay.

Microsatellite Instability (MSI) refers to the occurrence of a new microsatellite allele due to any change in the length of a microsatellite caused by the insertion or deletion of a repeating unit in a tumor compared with normal tissue. MSI status in this study was detected by NGS or IHC staining for mismatch repair proteins.

### Statistical analysis

All data management and statistical analysis were completed using GraphPad Prism software version 5.0 and R version 3.6.1. Depending on the DDR mutation status, these patients were separated into mutated and wild-type groups. Baseline characteristics were compared between the two groups using the Pearson chi-square test for categorical variables and Student t-test for continuous variables. And the respective correlation of DDR gene mutation and TMB, PD-L1 expression, or MSI status were analyzed with the t-test. The Kaplan–Meier method and log-rank test (Mantel-Cox) were used to compare the differences in OS between different groups. The R package “survival” was used to perform the Kaplan–Meier curves. The “ggplot2” and “forest plot” packages were used for graph production. Cox proportional hazard analysis was used to identify which were correlated with the prognosis of PDAC among clinical characteristics and DDR genes. For this study, P ≤ 0.05 was considered statistically significant.

## Results

### Patient characteristics

A total of 195 PDAC patients from multiple medical centers in China were enrolled in this retrospective study. The vast majority of patients were recruited from West China Medical Center between January 2016 and December 2019, and the rest came from the Cancer Hospital of Fudan University and the People's Hospital of Sichuan Province. Demographics and clinicopathological data of the study population were listed in Table 1. The median age of all patients was approximately 60 years (range: 27–79 years), and males were moderately overrepresented compared with females (56.4% vs 43.6%). A family history of any malignancy in first-degree relatives was noted in 31 patients (15.9%). 109 resectable patients (55.9%) and 85 unresectable patients (43.6%) received curative surgery and palliative surgery or just biopsy, respectively. At any point during the disease, 60 patients (30.8%) eventually had signs of metastasis. Limited-stage patients accounted for 36.7% of overall patients and 123 patients with advanced disease (62.8%) were included in this study.

**Table 1:** Baseline characteristics of overall patients

Variable	Overall cohort, N = 195	DDR status		
		mut N = 30 (15.4%)	wt N = 165 (84.6%)	p value
Age at diagnosis				0.35
Median, years	59.3	59.6	59.2	
Sex, n (%)				0.569
Male	110 (56.4%)	15 (50%)	95 (57.6%)	
Female	85 (43.6%)	15 (50%)	70 (42.4%)	
Family history of cancer, n (%)	31 (15.9%)	7 (23.3%)	24 (14.5%)	0.347
Pancreatic cancer	5 (2.6%)	1 (3.3%)	4 (2.4%)	
Any cancer	26 (13.3%)	6 (20%)	20 (12.1%)	
Location of primary tumor, n (%)				0.253
Head/uncinate	123 (63.1%)	15 (50%)	108 (65.5%)	
Body/tail	47 (24.1%)	10 (33.3%)	37 (22.4%)	
NA	24 (12.3%)	5 (16.7%)	19 (11.5%)	
Surgery, n (%)				0.674
R0 (Negative margin)	109 (55.9%)	16 (53.3%)	93 (56.4%)	
R1 (Positive margin)	83 (42.6%)	13 (43.3%)	70 (42.4%)	
NA	3 (1.5%)	1 (3.3%)	2 (1.2%)	
Pathological T stage, n (%)				0.596
T1 and T2	68 (34.9%)	9 (30.0%)	59 (35.8%)	
T3 and T4	124 (63.6%)	20 (66.7%)	104 (63.0%)	
NA	3 (1.5%)	1 (3.3%)	2 (1.2%)	
Pathological N stage, n (%)				0.47
N0	85 (43.6%)	15 (50.0%)	70 (42.4%)	
N1/N2	107 (54.9%)	14 (46.7%)	93 (56.4%)	
NA	3 (1.5%)	1 (3.3%)	2 (1.2%)	
Metastasis, n (%)				0.632
M0	132 (67.7%)	19 (63.3%)	112 (67.9%)	
M1	60 (30.8%)	10 (33.3%)	50 (30.3%)	
NA	3 (1.5%)	1 (3.3%)	2 (1.2%)	
Stage, n (%)				0.950
Limited stage	72 (36.7%)	11 (36.7%)	61 (37.0%)	
Advanced stage	123 (62.8%)	19 (63.3%)	104 (63.0%)	
Perivascular invasion, n (%)				0.686
Present	40 (20.5%)	6 (20.0%)	34 (20.6%)	
Absent	152 (77.9%)	23 (76.7%)	129 (78.2%)	
NA	3 (1.5%)	1 (3.3%)	2 (1.2%)	
Perineural invasion, n (%)				0.68
Present	75 (38.5%)	11 (36.7%)	64 (38.8%)	
Absent	117 (60.0%)	18 (60.0%)	99 (60.0%)	
NA	3 (1.5%)	1 (3.3%)	2 (1.2%)	
CA 19–9, n (%)				0.055
Normal	44 (22.6%)	7 (23.3%)	37 (22.4%)	
Elevated	145 (74.4%)	20 (66.7%)	125 (75.8%)	
Unknown	6 (3.1%)	3 (10.0%)	3 (1.8%)	
Surgery, n (%)				0.857
Curative surgery	109 (55.9%)	16 (53.3%)	93 (56.4%)	
Unresectable cancer	85 (43.6%)	14 (46.7%)	71 (43.0%)	
NA	1 (0.5%)	0 (0.0%)	1 (0.6%)	
Adjuvant radiotherap or chemotherapy, n (%)				0.094
Yes	84 (43.1%)	15 (50%)	69 (41.8%)	
No	44 (22.5%)	7 (23.3%)	37 (22.4%)	
Unknown	67 (34.4%)	8 (26.7%)	59 (35.8%)	

*mut* mutant, *wt* wild-type, *CA* carbohydrate antigen, *DDR* DNA damage repair, *N* node, *N* number, *T* tumor

P value was calculated by x^2^ except t test for age

In total, 30 patients (15.4%) were identified as mutant germline or somatic DDR gene in our study by NGS. The remaining 165 patients (84.6%) were therefore confirmed as DDR wild-type genotype and were matched by several clinical characteristics to the DDR mutated patients (Table 1). Generally, no significant difference in baseline characteristics of patients between the two groups. For example, there were equal numbers of male and female patients in DDR mutated groups, and male patients were slightly overrepresented in the wild-type group (50% DDR mut vs 57.6% DDR wt, p = 0.569). The percentage of a family cancer history was similar in each group, also for the presence of pancreatic cancer (3.3% DDR mut vs 2.4% DDR wt, p = 0.347). Regarding the TNM staging, the composition of each stage of patients was similar between the two groups (T1-T2: 30.0% DDR mut vs 35.8% DDR wt; T3-T4 66.7% DDR mut vs 63.0% DDR wt; p = 0.596). More importantly, there was no difference between patients in limited-stage or advanced stage (p = 0.950). In conclusion, these results showed that no significant difference in other variables between the two groups, except for the DDR gene mutation status.

### Mutation profiles of main driver genes

**Fig. 1:**
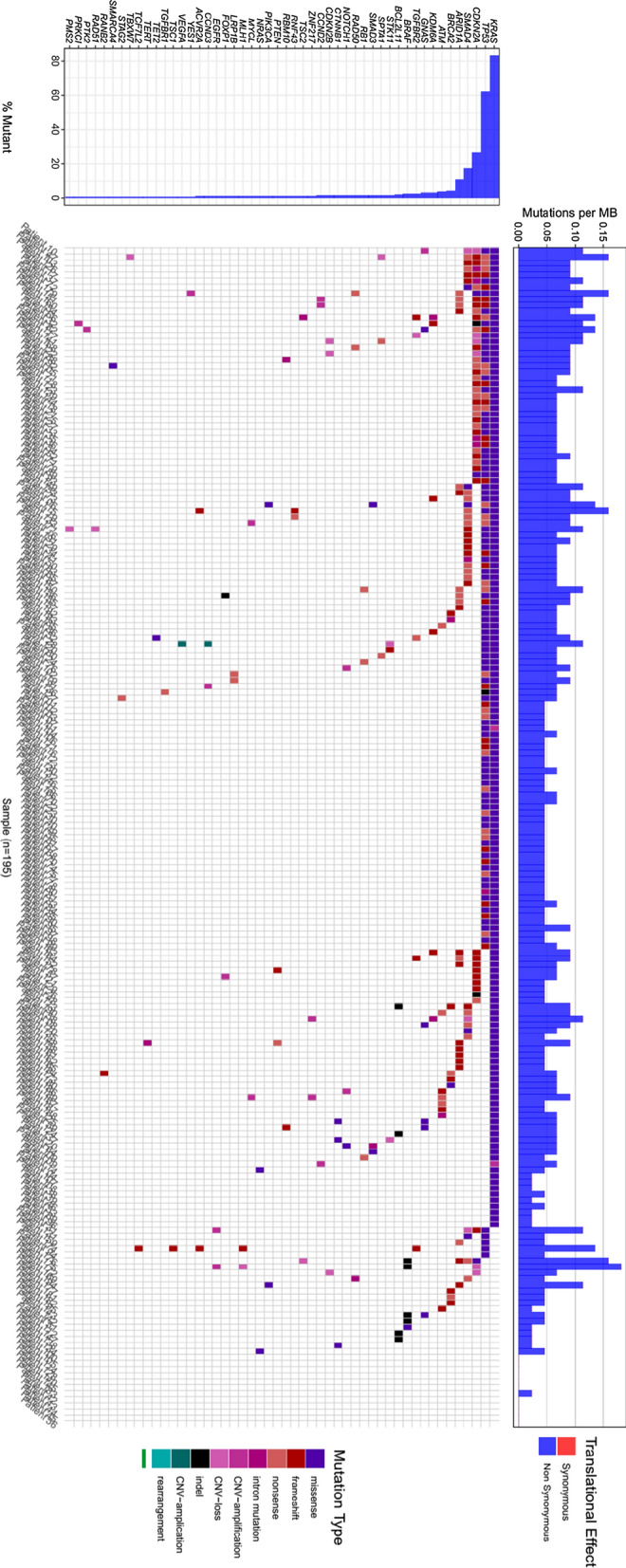
Mutation landscape of the 195 Chinese PDAC patients in our study

**Fig. 2:**
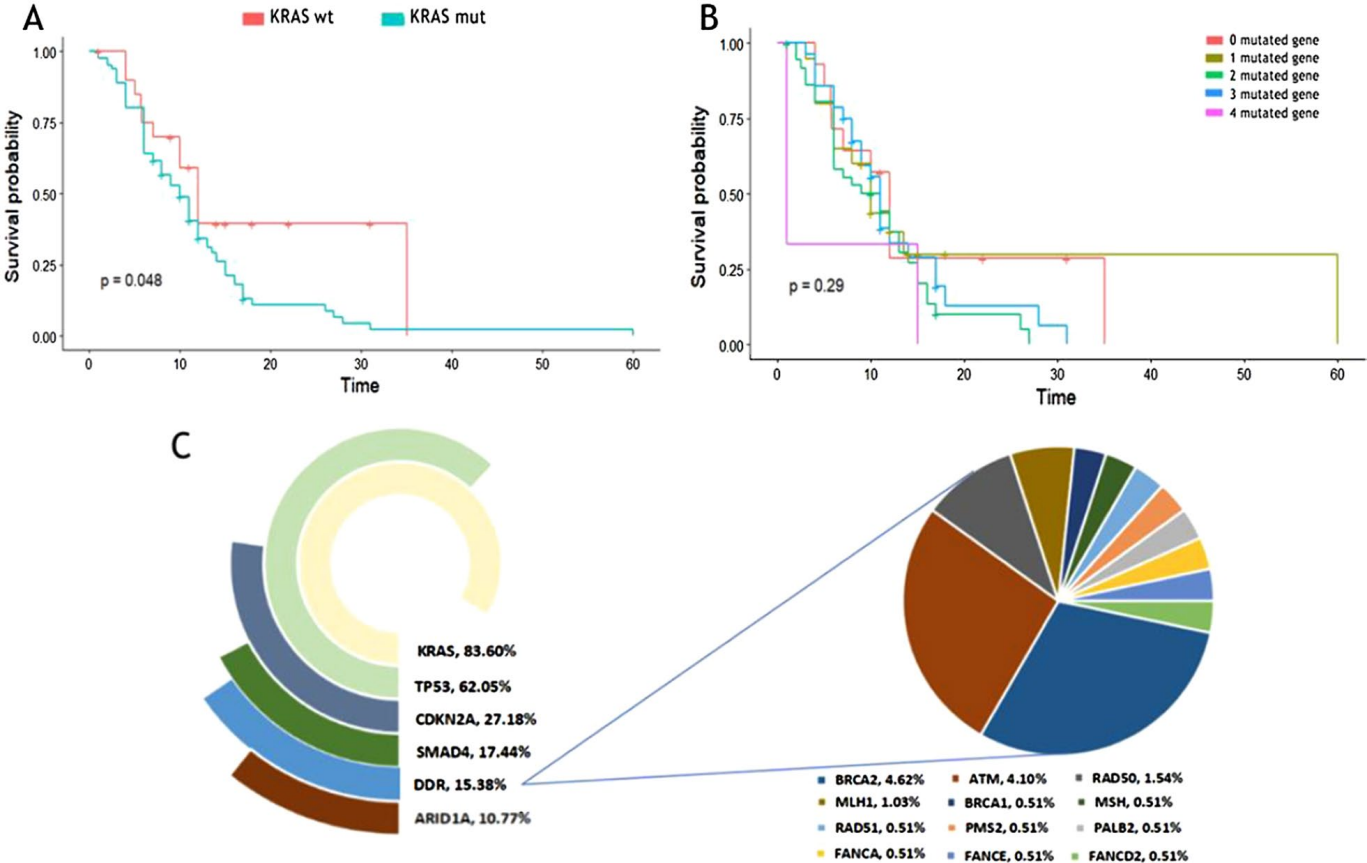
The association between driver genes and prognosis and the mutational landscape of DDR gene in our study. **A**The overall survival (OS) was shorter in advanced patients with KRAS mutation compared to those without. **B** The K-M survival analysis of advanced patients with 0 to 4 mutated driven gene. **C** The most frequently altered mutation genes and the percentage of mutations in DDR gene systems

We performed NGS for 195 Chinese PDAC patients enrolled in our study, which has revealed a complex mutational landscape about genes known to be important in pancreatic cancer. 565 deleterious mutations were detected in all patients. The average mutations per cancer sample were 2.9 and 13 patients (6.6%) did not have any alterations in the genes of our panel. The whole mutation landscape of our cohorts is illustrated in Fig. 1. Kirsten-ras protein (KRAS) was the most prevalent mutating gene, which occurred in 83.6% of patients of our cohorts. Other frequent genomic alterations were listed as follows: tumor protein p53 (TP53) (62.05% in our cohorts vs 51% in TCGA), cyclin-dependent kinase inhibitor 2A (CDKN2A) (27.18% vs 11%), drosophila mothers against decapentaplegic homolog 4 (SMAD4) (17.44% vs 15%). Next, we investigated the influence of mutations in driver genes on the clinical outcomes of advanced patients. KRAS mutated patients with significantly lower overall survival (OS) than wild-type patients (Fig. 2A). Interestingly, in this study, no significant correlation was found between the number of drive gene mutations and OS (Fig. 2B), which differed from the report of other studies that more drive gene mutations may lead to shorter survival in PDAC patients [16, 17]. As the fifth most common genetic alteration, a handful of genes related to DNA damage repair were identified in 15.38% of patients in our study. Among the genetic alterations, BRCA2 germline mutation was the most prevalent mutation of DDR deficiency. BRCA2, ATM, RAD50 and MLH1 genes were mutated in 9 (4.62%), 8 (4.10%), 3 (1.54%) and 2 (1.03%) of all patients, respectively. Other mutant DDR genes, such as BRCA1, MSH, RAD51, PMS2, PALB2, FANCA, FANCE, BLM, CHEK2, and FANCD2, were found in one patient (0.51%), respectively (Fig. 2C).

### Survival analyses based on DDR mutation status

**Fig. 3:**
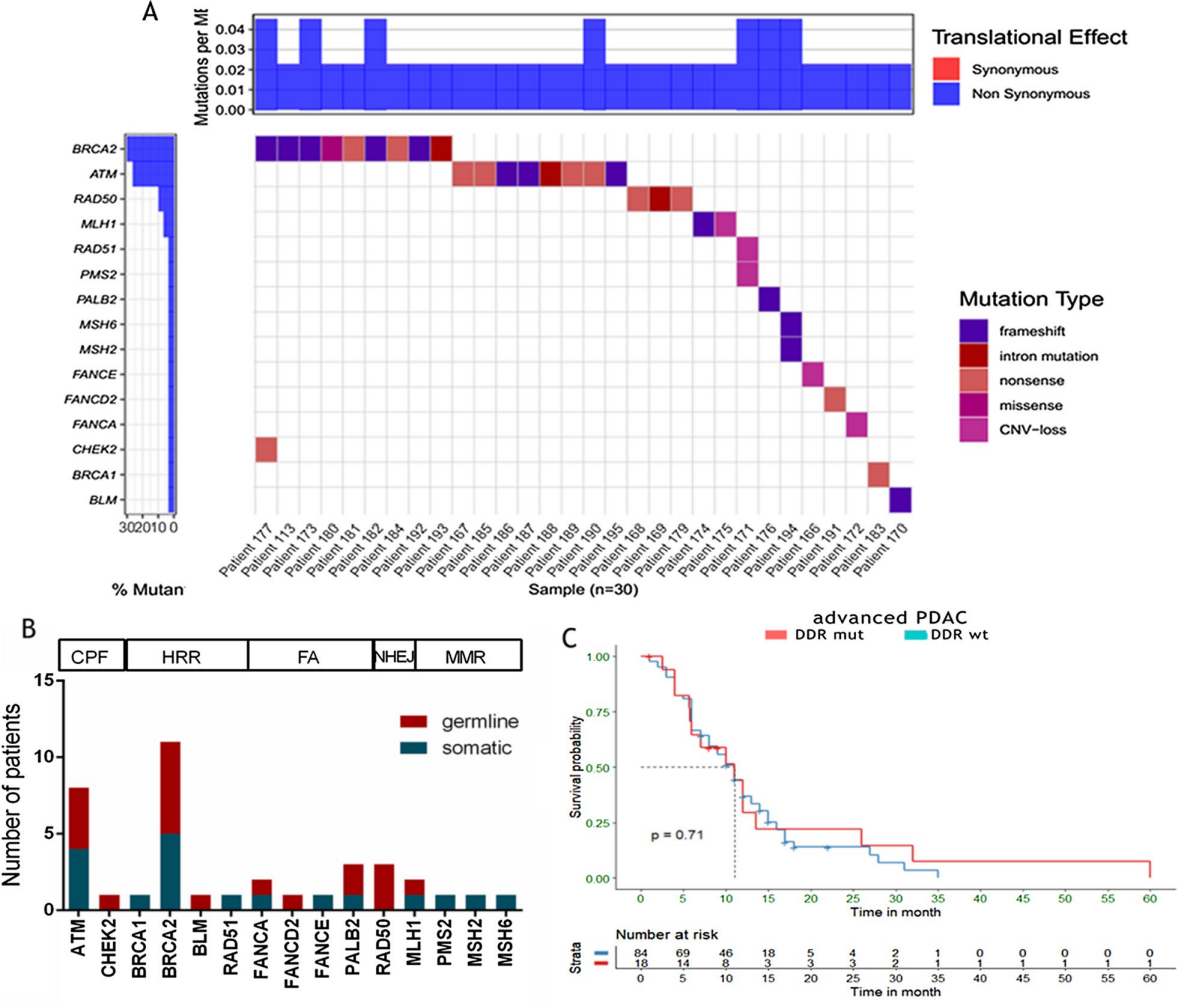
The mutation profile of DDR mutation in our cohorts and the correlation of DDR mutation status and overall survival of patients. **A** Mutation profile of DDR gene mutations in 30 patients. **B** The distribution and numbers of germline and somatic gene mutations in each individual sub-pathways of DDR systems. **C** The overall survival of advanced patients with DDR deficiency and those with intact DDR genes

The mutation profiles of all DDR gene mutations detected in our study has shown in Fig. 3A. There were several different alteration types among these mutations, including missense, nonsense, frameshift, intron mutation, and copy number variation (CNV)-loss. The detailed mutational information (mutation level, amino acid change, and corresponding functions) was listed in Table 2. A total of 36 mutations of DDR genes were identified in 30 patients, including 19 somatic mutations and 17 germline mutations (Fig. 3B). Six (3.07%) patients had more than one mutation. We recorded 12 germline and somatic deleterious BRCA1/2 mutations in 9 patients, 1 of which (0.5%) occurred in BRCA1, and 11 (4.7%) occurred in BRCA2. Among them, 2 patients had 2 sites of BRCA2 mutation simultaneously.

**Table 2:** Mutation details of DNA damage repair gene in 30 patients of our cohort

Patient ID	Sex	Age	Mut Level	Amino acid change	Function	Detection Panel
Patient 26	M	55	ATM germline	p.R1882*	Nonsense	417-gene
Patient 87	M	61	ATM germline	p.C1899*	Nonsense	417-gene
Patient 100	F	71	ATM germline	p.K468Efs*18	Frameshift	150-gene
Patient 135	F	71	ATM germline	p.Q441Afs*45	Frameshift	417-gene
Patient 195	M	55	ATM somatic	p.K2811Sfs*46	Frameshift	417-gene
Patient 140	F	63	ATM somatic	R1898*	Nonsense	417-gene
Patient 119	F	69	ATM somatic	p.G509*, p.L1651*	Nonsense	150-gene
Patient 42	M	63	ATM somatic	c.2921 + 1G > A	Intron mutation	381-gene
Patient 25	F	54	BLM germline	p.L258Efs*7	Frameshift	417-gene
Patient 186	F	45	BRCA1 somatic	p.R1443*	Nonsense	417-gene
Patient 11	F	67	BRCA2 somatic	p.T3033Nfs*11, p.K437Ifs*22	Frameshift	417-gene
Patient 130	F	34	BRCA2 somatic	p.R3128*	Nonsense	150-gene
Patient 143	M	63	BRCA2 somatic	D2723N	Missense	381-gene
Patient 162	M	55	BRCA2 somatic	p.R2494*	Nonsense	150-gene
Patient 130	F	34	BRCA2 germline	p.N2137Kfs*29	Frameshift	150-gene
Patient 193	M	55	BRCA2 germline	c.3847_3848del	Indel	417-gene
Patient 124	F	49	BRCA2 germline	Y1894*	Nonsense	381-gene
Patient 180	F	73	BRCA2 germline	p.Q1073Rfs*4	Frameshift	417-gene
Patient194	M	34	BRCA2 germline	p.V1283Kfs*2	Frameshift	417-gene
Patient 63	F	68	FANCA somatic	–	CNV-amplification	417-gene
Patient 86	F	79	FANCE somatic	–	CNV-loss	417-gene
Patient 75	M	60	FANCD2 germline	p.Q718*	Nonsense	417-gene
Patient 191	M	47	MLH1 somatic	p.N287Kfs*10	Frameshift	150-gene
Patient 145	M	57	MLH1 somatic	–	CNV-loss	150-gene
Patient115	M	62	MSH2 somatic	p.N566Ifs*24	Frameshift	150-gene
Patient 115	M	62	MSH6 somatic	p.F1088Sfs*2	Frameshift	150-gene
Patient 192	F	50	PALB2 somatic	p.F440Lfs*12	Frameshift	417-gene
Patient 192	F	50	PALB2 germline	p.R753*	Nonsense	417-gene
Patient 50	F	56	PMS2 somatic	–	CNV-loss	381-gene
Patient 160	M	51	RAD50 germline	p.Q826*	Nonsense	417-gene
Patient 28	M	67	RAD50 germline	c.3618 + 1G > A	Intron mutation	417-gene
Patient 152	M	76	RAD50 germline	p.R1077*	Nonsense	417-gene
Patient 50	F	56	RAD51 somatic	–	CNV-loss	381-gene

Survival analyses were conducted to confirm the predictive and prognostic value of mutations in DDR-related genes. In our cohort, there were 123 patients (63.1%) in the advanced cohorts and 102 of advanced patients had survival data. Among them, 104 patients were DDR wildtype, while 19 patients were identified as DDR deficiency. The median OS of advanced patients was 11.69 months. The patients with DDR deficiency showed no benefit in OS compared to wild-type patients (p = 0.71) (Fig. 3C).

**Fig. 4:**
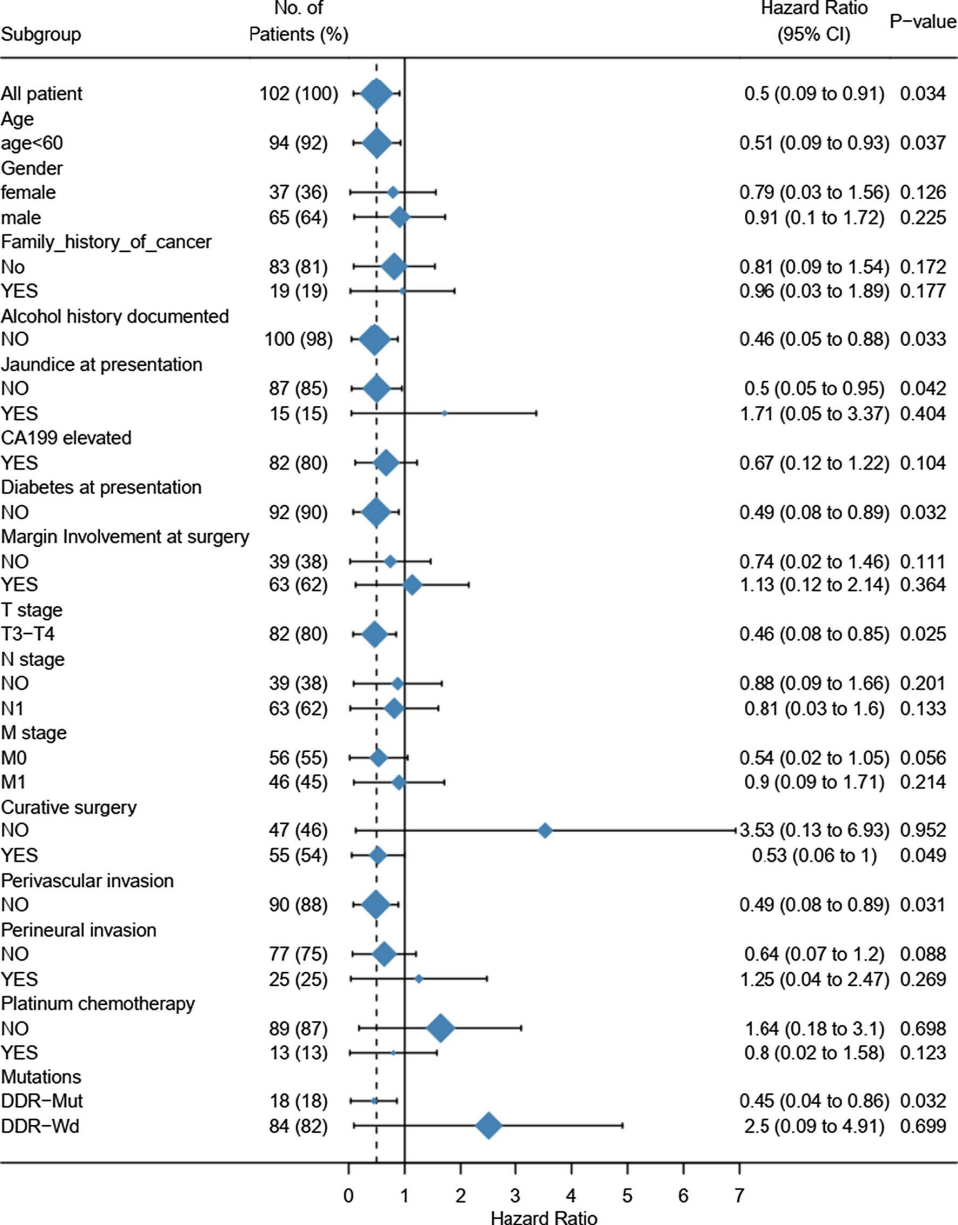
The forest plot for cox multivariate analysis of 102 advanced PDAC patients in our study

Besides, we also made a forest plot for a cox multivariate analysis of 102 advanced PDAC patients in our study (Fig. 4). As the results showed, age less than 60-year-old, alcohol history, jaundice or diabetes at presentation, T stage, adoption of curative surgery, perivascular invasion, and DDR mutation could act as the independent prognostic factors. Except for the influence of other covariates, DDR mutation could predict the prognosis of PDAC patients (HR 0.04–0.86, P = 0.032).

### The effects of olaparib, platinum-based chemotherapy and PD-1/PD-L1 blockade on overall survival to patients with the DDR deficiency

**Fig. 5:**
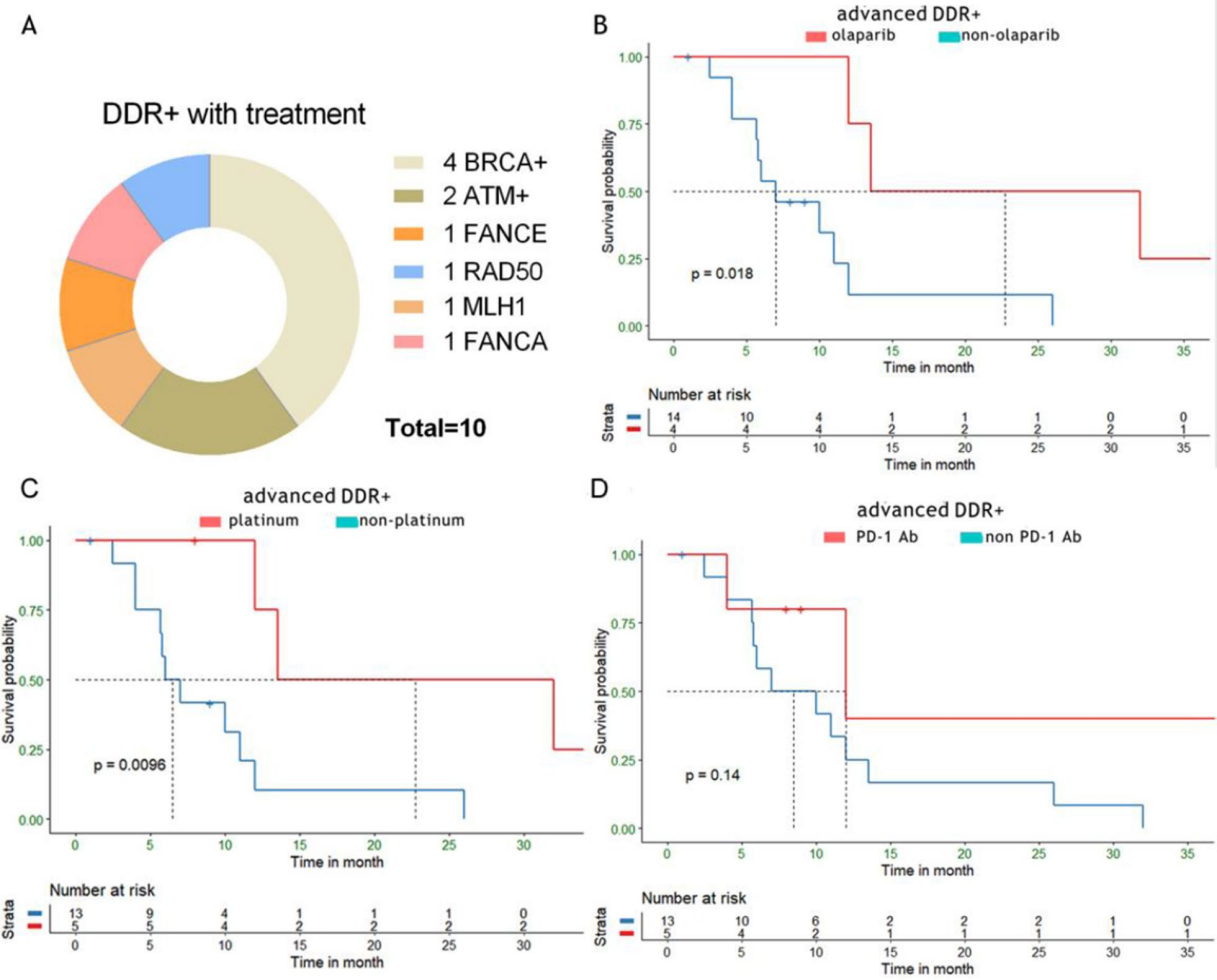
The respective relationship between OS and olaparib, platinum-based chemotherapy or PD-L1 blocking therapy in defective DDR patients. **A** The constitution of mutant DDR genes in patients with any one treatment of olaparib, platinum-based chemotherapy and PD-1/PD-L1 blockade. **B** In the subgroup of locally advanced or metastatic PDACs, there were significant difference between defective DDR patients with or without olapatib treatment. **C** Application of platinum-based chemotherapy positively correlated with the prolonged OS in locally advanced or metastatic PDAC patients. **D** The difference of OS between locally advanced or metastatic patients who treated with PD-L1 blockade and those without

Of all the 195 patients, 22 have ever received any one of these DDR targeting drugs (olaparib, platinum-based chemotherapy, and PD-1 blockades), and ten of them harbored DDR deficiency. Most patients who received these drugs harbored BRCA1/2 or ATM mutations (Fig. 5A). In the 18 advanced DDR-mutated patients, 4 patients received the second-line olaparib treatment after the failure of chemotherapy with gemcitabine and nab-paclitaxel or platinum. An improvement of OS was observed in the group with olaparib treatment compared to those without (p = 0.034; Fig. 5B).

There were overall 15 patients treated with platinum-based chemotherapy in our study, 9 of them were DDR wild-type while 6 were in the DDR mutated group. In advanced patients with DDR mutations, a total of 5 patients have received platinum chemotherapy during the whole therapeutic course. 3 of them received second-line platinum-based chemotherapy, including 1 patient with mFOLFIRINOX (modified 5-Fluorouracil, leucovorin, irinotecan, and oxaliplatin) regimen, after the tumor progression of gemcitabine plus nab-paclitaxel. The other two patients both received gemcitabine and platinum chemotherapy as the first-line treatment. However, one patient had progressive disease after 3 cycles of platinum chemotherapy and another patient had the disease recurrence in two years. In the advanced patients, platinum-based chemotherapy was also found to result in favorable OS (p = 0.0096, Fig. 5C). Next, we investigated the correlation between DDR deficiency and response to PD-1 inhibitors. Although PD-L1 overexpressed in tumors of 6 advanced patients, the efficacy of PD-1 blockades was a little disappointing: 1 patient with intact DDR genes had stable disease (SD), meanwhile, of the remaining 5 patients with DDR deficiency, 1 was evaluated as partial response (PR), 3 as SD, and 1 as progression disease (PD) (based on RECIST 1.1). However, in the advanced patients with DDR deficiency, the OS was not significantly prolonged after the treatment of PD-1 blockades (p = 0.14; Fig. 5D).

**Fig. 6:**
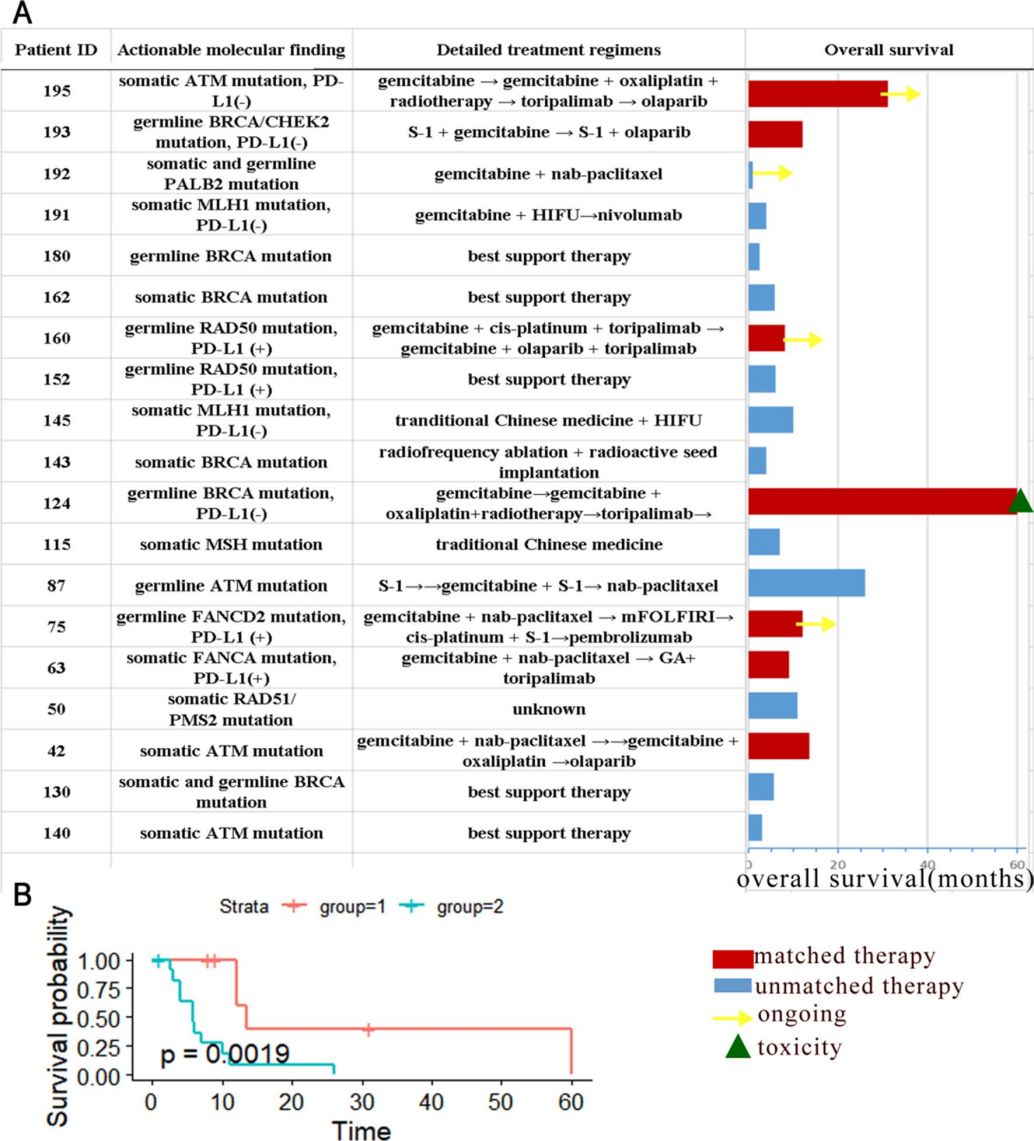
Actionable alterations combined with overall survival for molecularly matched and unmatched therapies. **A** The molecular mutations and therapeutic regimens in advanced patients with DDR deficiency and detailed clinical and survival data. **B** Matched therapy significantly improved the OS of advanced patients with DDR mutations than those with unmatched therapy. Matched therapy was defined as precise treatment according to the molecular profiling of the individual patient (olaparib and platinum-based chemotherapy for DDR mutations, PD-1 blockade for positive PD-L1 expression). FOLFIRI: flurouracil and irinotecan. FOLFIRINOX: fluorouracil, irinotecan, and oxaliplatin. HIFU: high intensity focused ultrasound

13 advanced patients with DDR deficiency had the treatment and survival records. Detailed data of these individual patients were summarized in Fig. 6A. Matched therapy was defined as precise treatment according to the molecular profiling of the individual patient. For example, the matched therapy of DDR mutations included olaparib and platinum-based chemotherapy, and PD-1 blockade was matched therapy for positive PD-L1 expression. As shown in Fig. 6B, the participation of molecularly matched therapy in the treatment course significantly improved the overall survival of patients compared to those treated with unmatched therapy.

### Correlations between hypermutation phenotype and DDR mutation

**Fig. 7:**
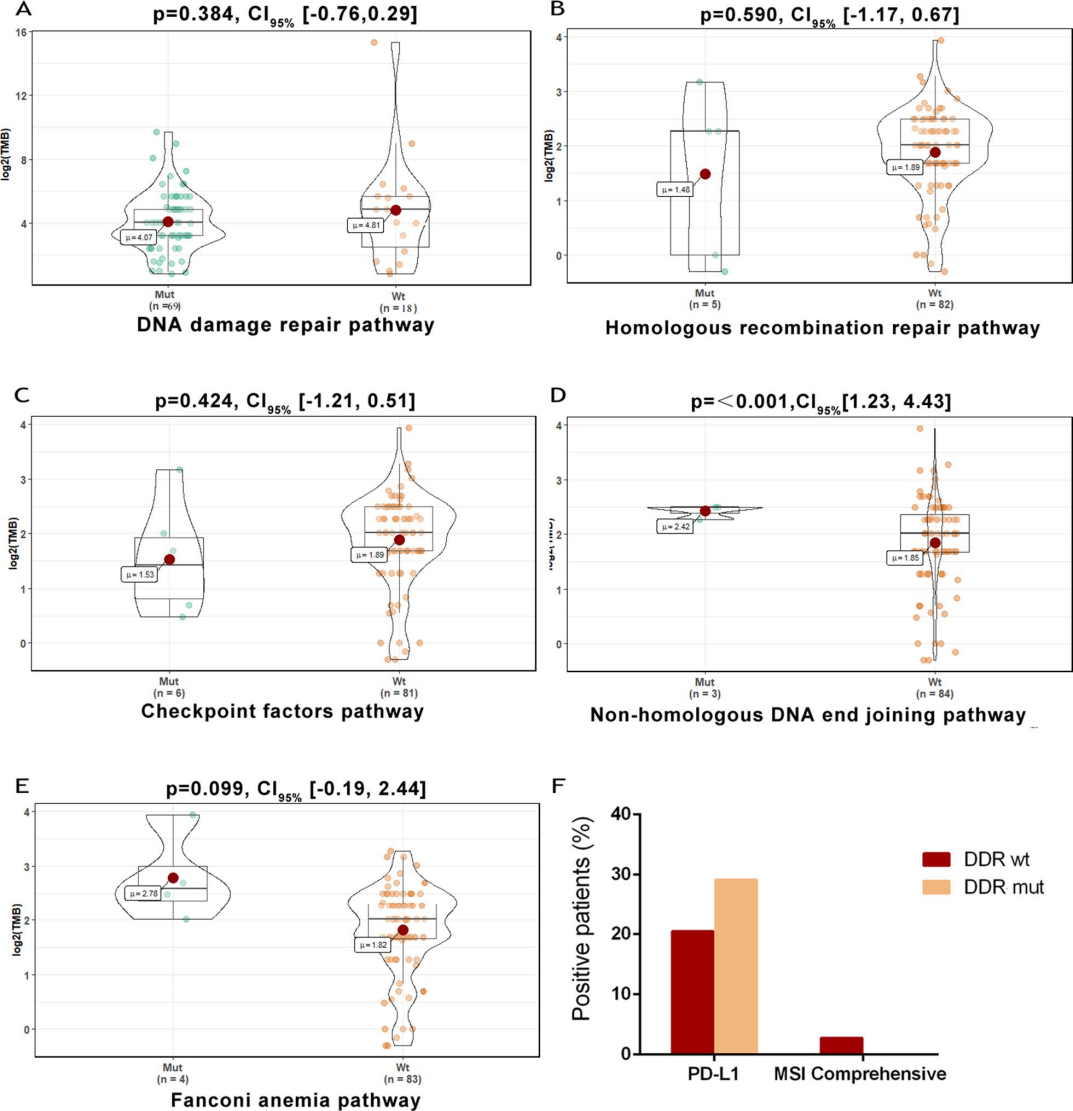
Distribution of TMB based on DNA damage-related gene-set alteration. **A** The relationship between TMB and the DDR gene status. **B**–**E** Comparison of the TMB levels between patients with the mutation of respective subways of DDR systems, HRR (**B**), CPF (**C**), NHEJ (**D**), and FA (**E**), and those without corresponding mutations. The y axis is indicating TMB per megabase in log2 scale. **F** Patients with DDR deficiency harbored higher percentage of PD-L1 overexpression compared to those with DDR wild-type. Abbreviations: DDR, DNA damage repair; FA, Fanconi anemia; HR, homologous recombination; NHEJ, non-homologous end joining; CPF, checkpoint factors; TMB, tumor mutation burden

In our study, TMB could be evaluated in 87 patients who profiled by the 417-gene panel. The median level of TMB was 4.9 mutations/Mb (range, 0.81–15.32 mutations/Mb). By analyzing the sequencing data of enrolled patients, we identified no significant difference of TMB between patients with DDR mutations and those in wild-type status (P = 0.384; Fig. 7A). However, in the DDR mutated group, a higher proportion of patients had a medium or high level of TMB (56.25% DDR mut vs 38.23% DDR wt), and fewer patients were located at the low level of TMB (31.25% DDR mut vs 47.06% DDR wt).

To meet the need to respond appropriately to different kinds of DNA damage, mammalian cells have evolved intricate DNA repair pathways to repair a large variety of structurally genotoxic damages: mismatch repair (MMR), base-excision repair (BER), nucleotide excision repair (NER), homologous recombination (HR), non-homologous DNA end joining (NHEJ) pathway, translesion synthesis (TLS), Fanconi anemia (FA) and checkpoint factors (CPF). In this study, the mutational genes were associated with five pathways (Fig. 3B). To further disclose the main contributing components affecting the connection between DDR mutations and TMB, we investigated whether the mutations among these pathways of the DDR system may affect the TMB levels. As shown in Fig. 7B–E, patients with genetic alterations in CPF (p = 0.424), HRR (p = 0.590), and FA pathways (p = 0.099) failed to show significant differences with corresponding wide-type patients. However, NHEJ pathway alterations demonstrated a comparably higher level of TMB than the NHEJ wild-type groups (p < 0.001).

In our study, 89 of 195 patients had the available information of microsatellite status. One patient was evaluated as MSI-high by known NGS sequencing sites and another MSI-low was confirmed by immunohistochemistry (IHC) detection. And the remaining patients were all microsatellite stable (MSS). In contrast to our hypothesis, the two MSI patients were both DDR wild-type. IHC information of PD-L1 protein was available in 102 patients, and 23 of them (22.5%) were positive. In the DDR mutated group, the proportion of patients with PD-L1 overexpression was a little higher than that in the wild-type group (29.17% DDR mut vs 20.51% DDR wt) (Fig. 7F).

## Discussion

To our knowledge, this retrospective study is the first study focusing on the germline or somatic DDR mutations of PDAC patients in the Chinese population.

First of all, the top 4 commonly mutated genes between different racial populations were almost the same: KRAS, TP53, CDKN2A, and SMAD4, which was in accord with the recent study conducted in Chinese patients [18] and some previous studies about western PDAC patients [19, 20]. However, different racial cohorts may have different tendencies of the fifth most frequent mutation. ARID1A was supported by some research to be the fifth most altered gene with a more than 10% incidence rate [21]. A whole-genome sequencing conducted in 100 Australian PDAC patients showed that ARID1A mutation was prevalent, which was consistent with our cohorts [5]. And other candidates including FLG [22] and myeloid/lymphoid or mixed-lineage leukemia protein 3 (MLL3) [23]. ARID1A, however, was not listed as the top 5 mutated genes in the current understanding of PDAC molecular type [24]. Despite the molecular status of main driver genes being considered to potentially influence OS in some studies [22, 25], in our research, there was no survival difference between patients with or without any mutations of the top 4 mutated genes except KRAS. KRAS mutation also was confirmed to shorten the survival term of PDAC patients by other studies [16]. And the phenomenon that patients with altered driven genes may have worse OS, which was reported in other studies [17, 26], was not observed in our research.

The overall incidence of DDR mutation in our cohort was 15.38%, which is a little higher than what has been reported in other NGS studies: Wang et al. conducted a study of 540 Chinese PDAC patients and found that germline mutations were identified in 60 patients (11.1%) [18]. Matthew B et al. enrolled 289 resected PDAC patients of the USA and found that 7.3% carried the germline variants in 24 detected DDR genes [27]. A large-scale study of targeted genomic profile analyses showed that BRCA and FANC mutations were detected in 14% of 3594 international PDAC patients [20]. This figure was similar to the 17% prevalence of the DDR mutations in the high-risk population (Ashkenazi Jews) [28]. Although further validations in a larger-scale population were required, it is hypothetical that the Chinese population was also at high risk for overall DDR mutations. The results emphasized that the DDR gene mutations were relatively common in Chinese PDACs, which required us to pay more attention.

Secondly, the clinical outcomes of DDR mutations were controversial. In the western cohorts, the OS of patients carried with BRCA1/2 or PALB2 mutation was prolonged compared to that of non-carriers (21.8 months DDR mut vs 8.1 months DDR wt) [29]. However, some studies showed that there was no prognostic difference between the two groups, and others even suggested that germline BRCA mutation may induce a worse prognosis [30]. No significant difference in OS was observed between the patients with or without DDR mutations in our study. The inconsistency of results may ascribe to the different characteristics of the enrolled population and the different therapeutic regimens they received.

Next, to further investigate the impact of DDR mutations as actionable genes to guide precision medicine. In advanced patients with DDR deficiency, we found that molecularly matched therapy significantly improved the OS of patients than those with unmatched therapy. The Know Your Tumor (KYT) trial also reported that the median PFS of patients with the matched therapy was significantly longer than the patients in the unmatched therapy group [31].

Additionally, no significant difference in TMB between patients with DDR mutations and those in wild-type status in our study. Of the four specific sub-pathways, mutation of the NHEJ pathway was the only one to induce higher TMB. In the DDR mutated group, a higher proportion of patients had a medium or high level of TMB and fewer patients were located at a low level of TMB. However, some studies found a positive correlation between TMB and DDR mutations. In Korean SCLC patients, a higher TMB level was identified in the DDR mutated group than the DDR wild-type group [32]. Another study revealed that deleterious alterations in 34 DDR related genes may exhibit high TMB levels and be independently related to better response to ICIs in metastatic urothelial cancer [33]. Furthermore, only 2 patients in our cohorts were identified with MSI-high but with the intact DDR genes. Contrary to other studies, DDR mutations were observed to positively correlate to MSI in a study [34]. These differences are mainly derived from a lot of absence of information on MSI status in our study. According to the latest ASCO clinical practice guideline, pembrolizumab is suggested for patients with MMR deficiency or MSI-high metastatic pancreatic cancer [35]. A study reported that PDAC patients with DDR mutations had a higher percentage of positive PD-L1 expression than wild-type [36]. In conclusion, the correlation between DDR mutations, MSI status, and PD-L1 expression needs to be further verified by large-scale research.

There were some limitations to this study. The first issue included controversial definitions of DDR genes and the relationship between these genes and their corresponding pathways. Due to insufficient knowledge about the intricate regulation mechanism of the DDR pathways, current research concerning PDAC used unrecognized standards to classify the DDR genes. For example, some studies defined 14 or 16 genes as the members of the DDR system [30, 37]. Patients in this study profiled using three different gene panels, which had specific probes covering a different range of gene sets. The different spectrum of detected genes may lead to the diverse classification of DDR mutations and affect the results of studies. Additionally, a low number of patients who received DDR related treatment limited the development of statistical analyses. Although there was a difference in median OS between two groups of small-sample comparison, it was still difficult to reach a significant P value according to statistic analyses. As a result, the lack of information and a small sample size may lead to some deviations in conclusions. Another limitation is the retrospective nature of our study. The impact of other treatment regimens the patients received, such as radioactive particle implantation, arterial infusion chemotherapy, or traditional Chinese medicine was beyond the scope of this study, which may lead to a large degree of therapeutic heterogeneity within the total cohorts. Even with these above-mentioned limitations, genomic data generated separately from some platforms validated our findings [21, 27, 38, 39]. Large-scale randomized controlled trials are expected to prospectively verify the predictive role of DDR mutations.

## Conclusions

In conclusion, our study described the mutation profiling of the currently largest Chinese PDAC population. The main driver genes of Chinese PDAC patients were KRAS, TP53, CDKN2A, and SMAD4. Patients with KRAS mutation showed worse OS than those without. DDR deficiency was identified in 15.38% of overall patients, mainly involving BRCA2, ATM, and RAD50 genes. Furthermore, our results portrayed a probably positive association between DDR mutations and the better therapeutic efficacy of olaparib and platinum-based chemotherapy in advanced PDAC patients. DDR mutations were limited in inducing a high mutation status of patients and higher sensitivity to PD-1 blockades. Our study provided a relatively comprehensive profile of DDR mutations in Chinese PDAC patients and suggested the potential connection between DDR mutation and therapeutic effects, which may catalyze further biomarker studies targeting impaired DNA pathways or immunotherapies.

## Supplementary Information

**Additional file 1:** Detailed List of the three panels used in our study.

## Data Availability

The datasets used and analyzed during the current study are available from the corresponding authors on reasonable request.

## References

[1] Siegel RL, Miller KD (2020). Cancer statistics. CA Cancer J Clin.

[2] Hruban RH, Gaida MM, Thompson E, Hong SM, Noë M, Brosens LA, Jongepier M, Offerhaus GJ, Wood LD (2019). Why is pancreatic cancer so deadly? The pathologist's view. J Pathol.

[3] Christenson ES, Jaffee E, Azad NS (2020). Current and emerging therapies for patients with advanced pancreatic ductal adenocarcinoma: a bright future. Lancet Oncol.

[4] Perkhofer L, Gout J, Roger E (2021). DNA damage repair as a target in pancreatic cancer: state-of the-art and future perspectives. Gut.

[5] Gupta M, Iyer R, Fountzilas C (2019). Poly(ADP-Ribose) polymerase inhibitors in pancreatic cancer: a new treatment paradigms and future implications. Cancers.

[6] Goldstein M, Kastan MB (2015). The DNA damage response: implications for tumor responses to radiation and chemotherapy. Annu Rev Med.

[7] O'Connor MJ (2015). Targeting the DNA damage response in cancer. Mol Cell.

[8] Basourakos SP, Li L, Aparicio AM, Corn PG, Kim J, Thompson TC (2017). Combination platinum-based and DNA damage response-targeting cancer therapy: evolution and future directions. Curr Med Chem.

[9] Campbell BB, Light N, Fabrizio D, Zatzman M, Fuligni F, de Borja R (2017). Comprehensive analysis of hypermutation in human cancer. Cell.

[10] Ferrone CR, Levine DA, Tang LH, Allen PJ, Jarnagin W, Brennan MF (2009). BRCA germline mutations in Jewish patients with pancreatic adenocarcinoma. J Clin Oncol.

[11] Holter S, Borgida A, Dodd A, Grant R, Semotiuk K, Hedley D (2015). Germline BRCA mutations in a large clinic-based cohort of patients with pancreatic adenocarcinoma. J Clin Oncol.

[12] Knijnenburg Theo A, Linghua W, Zimmermann Michael T (2018). Genomic and molecular landscape of DNA damage repair deficiency across the cancer genome atlas. Cell Rep.

[13] Haraldsdottir S, Hampel H, Tomsic J (2014). Colon and endometrial cancers with mismatch repair deficiency can arise from somatic, rather than germline, mutations. Gastroenterology.

[14] Su D, Zhang D, Chen K, Lu J, Wu J, Cao X (2017). High performance of targeted next generation sequencing on variance detection in clinical tumor specimens in comparison with current conventional methods. J Exp Clin Cancer Res.

[15] Richards S, Aziz N, Bale S (2015). Standards and guidelines for the interpretation of sequence variants: a joint consensus recommendation of the American College of Medical Genetics and Genomics and the Association for Molecular Pathology. Genet Med.

[16] Hu C, Hart SN, Polley EC, Gnanaolivu R, Shimelis H, Lee KY (2018). Association between inherited germline mutations in cancer predisposition genes and risk of pancreatic cancer. JAMA.

[17] Qian ZR, Rubinson DA, Nowak JA, Morales-Oyarvide V, Dunne RF, Kozak MM (2018). Association of alterations in main driver genes with outcomes of patients with resected pancreatic ductal adenocarcinoma. JAMA Oncol.

[18] Wang W, Zhou B, Ding Y (2020). The genomic features of Chinese pancreatic adenocarcinoma and the implications for therapy. Ann Oncol.

[19] Jones S, Zhang X, Parsons DW, Lin JC, Leary RJ, Angenendt P (2008). Core signaling pathways in human pancreatic cancers revealed by global genomic analyses. Science.

[20] Singhi AD, George B, Greenbowe JR, Chung J, Suh J, Maitra A (2019). Real-time targeted genome profile analysis of pancreatic ductal adenocarcinomas identifies genetic alterations that might be targeted with existing drugs or used as biomarkers. Gastroenterology.

[21] Lowery MA, Jordan EJ, Basturk O, Ptashkin RN, Zehir A, Berger MF (2017). Real-time genomic profiling of pancreatic ductal adenocarcinoma: potential actionability and correlation with clinical phenotype. Clin Cancer Res.

[22] Witkiewicz AK, McMillan EA, Balaji U, Baek G, Lin WC, Mansour J (2015). Whole-exome sequencing of pancreatic cancer defines genetic diversity and therapeutic targets. Nat Commun.

[23] Biankin AV, Waddell N, Kassahn KS, Gingras MC, Muthuswamy LB, Johns AL (2012). Pancreatic cancer genomes reveal aberrations in axon guidance pathway genes. Nature.

[24] Collisson EA, Bailey P, Chang DK, Biankin AV (2019). Molecular subtypes of pancreatic cancer. Nat Rev Gastroenterol Hepatol.

[25] owery MA, Jordan EJ, Basturk O, Ptashkin RN, Zehir A, Berger MF, (2017). Real-time genomic profiling of pancreatic ductal adenocarcinoma: potential actionability and correlation with clinical phenotype. Clin Cancer Res.

[26] Yachida S, White CM, Naito Y, Zhong Y, Brosnan JA, Macgregor-Das AM (2012). Clinical significance of the genetic landscape of pancreatic cancer and implications for identification of potential long-term survivors. Clin Cancer Res.

[27] Yurgelun MB, Chittenden AB, Morales-Oyarvide V, Rubinson DA, Dunne RF, Kozak MM (2019). Germline cancer susceptibility gene variants, somatic second hits, and survival outcomes in patients with resected pancreatic cancer. Genet Med.

[28] Salo-Mullen EE, O'Reilly EM, Kelsen DP, Ashraf AM, Lowery MA, Yu KH (2015). Identification of germline genetic mutations in patients with pancreatic cancer. Cancer.

[29] Kim A. Reiss, Shun Yu, Renae Judy, Heather Symecko, Katherine L. Nathanson, and Susan M. Domchek. Retrospective Survival Analysis of Patients With Advanced Pancreatic Ductal Adenocarcinoma and Germline BRCA or PALB2 Mutations. 10.1200/PO.17.00152 10.1200/PO.17.00152 35135099

[30] Sehdev A, Gbolahan O, Hancock BA, Stanley M, Shahda S, Wan J (2018). Germline and somatic DNA damage repair gene mutations and overall survival in metastatic pancreatic adenocarcinoma patients treated with FOLFIRINOX. Clin Cancer Res.

[31] Pishvaian MJ, Blais EM, Brody JR, Lyons E, DeArbeloa P, Hendifar A (2020). Overall survival in patients with pancreatic cancer receiving matched therapies following molecular profiling: a retrospective analysis of the Know Your Tumor registry trial. Lancet Oncol.

[32] Park S, Lee H, Lee B, Lee SH, Sun JM, Park WY (2019). DNA damage response and repair pathway alteration and its association with tumor mutation burden and platinum-based chemotherapy in SCLC. J Thorac Oncol.

[33] Teo MY, Seier K, Ostrovnaya I, Regazzi AM, Kania BE, Moran MM (2018). Alterations in DNA damage response and repair genes as potential marker of clinical benefit from PD-1/PD-L1 blockade in advanced urothelial cancers. J Clin Oncol.

[34] Tuli R, Shiao SL, Nissen N, Tighiouart M, Kim S, Osipov A (2019). A phase 1 study of veliparib, a PARP-1/2 inhibitor, with gemcitabine and radiotherapy in locally advanced pancreatic cancer. EBioMedicine.

[35] Sohal DPS, Kennedy EB, Khorana A, Copur MS, Crane CH, Garrido-Laguna I (2018). Metastatic pancreatic cancer: ASCO clinical practice guideline update. J Clin Oncol.

[36] Sherri Z. Millis , Brian L. Abbott , Erin H Baker , Ryan Bender , Jeffrey Swensen , Zoran Gatalica. Multiplatform molecular profiling of pancreatic adenocarcinomas to identify BRCA1/2 mutations and PD-1/PD-L1 status with therapeutic implications. 10.1200/jco.2015.33.15_suppl.4124

[37] Chae H, Kim D, Yoo C, Kim KP, Jeong JH, Chang HM (2019). Therapeutic relevance of targeted sequencing in management of patients with advanced biliary tract cancer: DNA damage repair gene mutations as a predictive biomarker. Eur J Cancer.

[38] Wartenberg M, Cibin S, Zlobec I, Vassella E, Eppenberger-Castori S, Terracciano L (2018). Integrated genomic and immunophenotypic classification of pancreatic cancer reveals three distinct subtypes with prognostic/predictive significance. Clin Cancer Res.

[39] Hu ZI, Shia J, Stadler ZK, Varghese AM, Capanu M, Salo-Mullen E (2018). Evaluating mismatch repair deficiency in pancreatic adenocarcinoma: challenges and recommendations. Clin Cancer Res.

