# Correction to: The germline/somatic DNA damage repair gene mutations modulate the therapeutic response in Chinese patients with advanced pancreatic ductal adenocarcinoma

**DOI:** 10.1186/s12967-021-02997-x

**Published:** 2021-08-12

**Authors:** Lin Shui, Xiaofen Li, Yang Peng, Jiangfang Tian, Shuangshuang Li, Du He, Ang Li, Bole Tian, Mao Li, Heli Gao, Ning An, Cheng Yi, Dan Cao

**Affiliations:** 1 Department of Abdominal Oncology, Cancer Center, State Key Laboratory of Biotherapy, West China Hospital, Sichuan University, Chengdu, China; 2 Department of Obstetrics and Gynecology, West China Second University Hospital, Sichuan University, Chengdu, China; 3 Department of Breast Surgery, The First Affiliated Hospital of Chongqing Medical University, Chongqing, China; 4 Department of Oncology, The Second Affiliated Hospital of Chengdu Medical College, China National Nuclear Corporation 416 Hospital, Chengdu, China; 5 Department of Pathology, West China Hospital, Sichuan University, Chengdu, China; 6 Pancreatic Surgery, West China Hospital, Sichuan University, Chengdu, China; 7 Department of Oncology, The Cancer Hospital of Fudan University, Shanghai, China; 8 Department of Oncology, The People‘s Hospital of Sichuan Province, Chengdu, China

## Correction to: J Transl Med (2021) 19:301https://doi.org/10.1186/s12967-021-02972-6

During the publication process the affiliations of this article [1] were not correctly updated. The updated affiliations are available via this correction article. The original article has been updated. We apologize for the inconvenience to the readers and authors.
